# Supplementary material for: Extracellular Vesicles Derived from Human Umbilical Cord Mesenchymal Stem Cells Attenuate Mast Cell Activation
Source: Antioxidants (Basel). 2022 Nov 17;11(11):2279. doi: 10.3390/antiox11112279 (PMC9686796; doi:10.3390/antiox11112279)
Supplement: Supplementary file 1 [file antioxidants-11-02279-s001.zip › antioxidants-1958976-supplementary 1.pptx]

## Slide 1
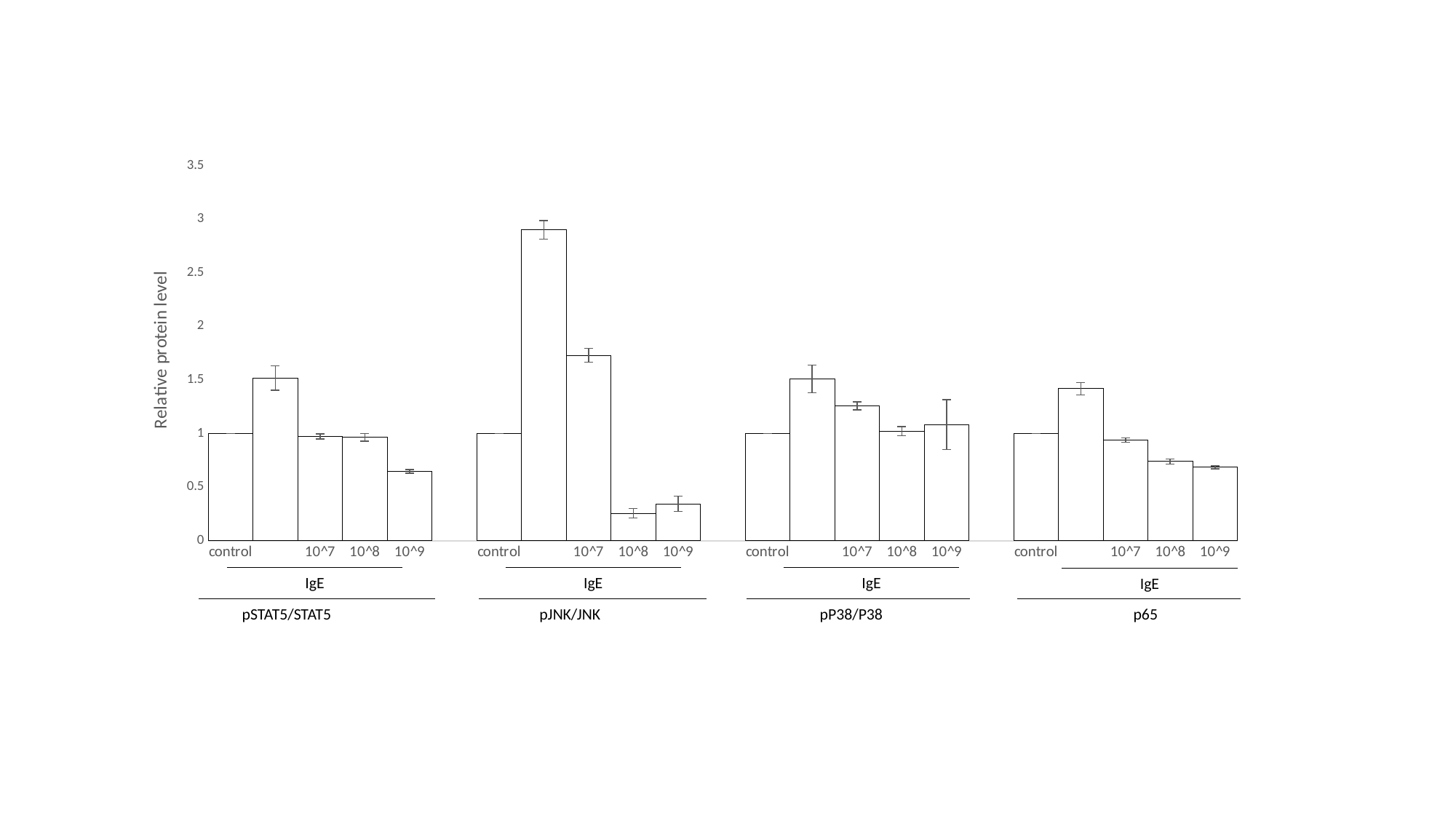

### Chart
| Category | |
|---|---|
| control | 1.0 |
| | 1.5176079748687836 |
| 10^7 | 0.972205025913409 |
| 10^8 | 0.9646428300085722 |
| 10^9 | 0.6464266623579036 |
| | None |
| control | 1.0 |
| | 2.9007280427208655 |
| 10^7 | 1.729962798839798 |
| 10^8 | 0.25502426344765733 |
| 10^9 | 0.34443430997591234 |
| | None |
| control | 1.0 |
| | 1.5082191964359655 |
| 10^7 | 1.2581701721545706 |
| 10^8 | 1.0216761124506937 |
| 10^9 | 1.08245604417743 |
| | None |
| control | 1.0 |
| | 1.4186836613766272 |
| 10^7 | 0.9385737399728692 |
| 10^8 | 0.7395505088972105 |
| 10^9 | 0.684563153267426 |IgE
IgE
IgE
IgE
pSTAT5/STAT5
pJNK/JNK
pP38/P38
p65
